# Integrative Analysis Revealing Human Heart-Specific Genes and Consolidating Heart-Related Phenotypes

**DOI:** 10.3389/fgene.2020.00777

**Published:** 2020-07-30

**Authors:** Jinsoo Ahn, Huiguang Wu, Kichoon Lee

**Affiliations:** ^1^Department of Animal Sciences, The Ohio State University, Columbus, OH, United States; ^2^College of Veterinary Medicine, Yangzhou University, Yangzhou, China

**Keywords:** heart-specific genes, differentially expressed genes, ingenuity pathway analysis, gene expression, heart disease

## Abstract

Elucidating expression patterns of heart-specific genes is crucial for understanding developmental, physiological, and pathological processes of the heart. The aim of the present study is to identify functionally and pathologically important heart-specific genes by performing the Ingenuity Pathway Analysis (IPA). Through a median-based analysis of tissue-specific gene expression based on the Genotype-Tissue Expression (GTEx) data, we identified 56 genes with heart-specific or elevated expressions in the heart (heart-specific/enhanced), among which three common heart-specific/enhanced genes and four atrial appendage-specific/enhanced genes were unreported regarding the heart. Differential expression analysis further revealed 225 differentially expressed genes (DEGs) between atrial appendage and left ventricle. Our integrative analyses of those heart-specific/enhanced genes and DEGs with IPA revealed enriched heart-related traits and diseases, consolidating evidence of relationships between these genes and heart function. Our reports on comprehensive identification of heart-specific/enhanced genes and DEGs and their relation to pathways associated with heart-related traits and diseases provided molecular insights into essential regulators of cardiac physiology and pathophysiology and potential new therapeutic targets for heart diseases.

## Introduction

During heart development, conserved transcriptional networks govern cardiac cell fate determination, cardiomyocyte differentiation, and cardiac morphogenesis (Olson, 2006). Identification of cardiac-specific genes and elucidation of their expression patterns are therefore crucial for understanding developmental, physiological, and pathological processes of the heart. Importantly, characteristic transcriptional profiles between atria and ventricles of the heart have been linked to heart region-specific differences in morphogenesis, structure, contractility, and electrophysiological properties (Bao et al., 1999; Barth et al., 2005; Olson, 2006). Based on these significances of abundant or differential expression of genes in the heart, comprehensive screenings of heart-specific genes and differentially expressed genes (DEGs) between heart chambers gain significant attention. To this end, the Genotype-Tissue Expression (GTEx) data can be analyzed to provide up-to-date RNA-seq transcriptomic profiling for various human tissues (GTEx Consortium, 2017). Recently, a robust approach of identifying tissue-specific genes using GTEx data was developed by our group (Ahn et al., 2019). This approach was further used to comprehensively identify heart-specific genes and DEGs in this study.

In order to obtain insight into functional relevance of genes of interests in organ development and various pathological conditions, molecular interactions and pathways under various developmental and experimental conditions can be analyzed by Ingenuity Pathway Analysis (IPA) (QIAGEN Inc.,^1^) as described in a previous publication (Kramer et al., 2014). Furthermore, previous studies documented a large number of candidate genes and loci associated with heart diseases and characteristics of the heart to determine genetic factors associated with physiology and pathophysiology of the heart (Buniello et al., 2019). However, despite these achievements, evidence has yet to be explored to link between genes expressed exclusively or abundantly in the heart (e.g., heart-specific genes and heart-enhanced genes described in the current study) and heart traits or diseases. The objectives of the current study were to identify heart-specific and heart-enhanced genes including functionally unreported novel genes, examine DEGs between atrial appendage and left ventricle, and provide an approach that can narrow down candidate genes for further research by performing IPA-based pathway analysis.

Herein, using gene expression values from atrial appendage and left ventricle of the heart, three novel protein-coding genes that are commonly specific or enhanced in both atrial appendage and left ventricle, four novel protein-coding genes that are specific or enhanced in atrial appendage, and 225 protein-coding DEGs between the two tissues were identified. By integrating data from pathway analysis, evidence of interrelationships between these genes and major heart-related traits or diseases including cardiogenesis, cardiac muscle contraction, fibrosis, heart failure, organismal death, morphogenesis of cardiac muscle, hypertrophy of left ventricle, and calcium signaling were consolidated. Overall, in this study, heart-specific genes and DEGs between atrial appendage and left ventricle and their relations with pathways associated with heart-related traits and diseases were comprehensively identified, providing molecular insights into essential regulators of cardiac physiology and pathophysiology and potential new therapeutic targets for heart diseases.

## Materials and Methods

### Data Collection and Filtering

**FIGURE 1:**
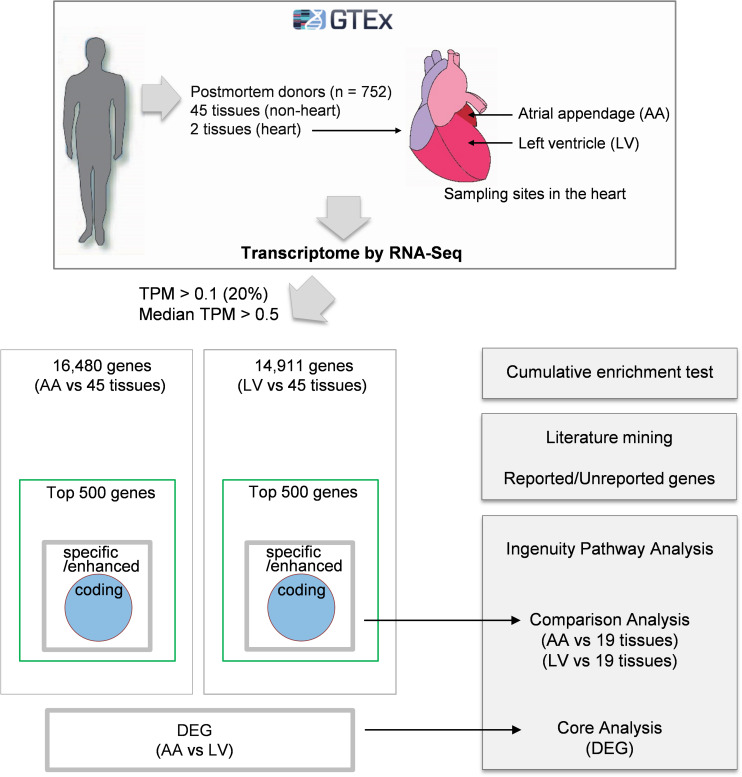
Schematic diagram of the approach. Gene expression values (TPMs) across 47 human tissues including atrial appendage (AA) and left ventricle (LV) were collected from the GTEx v7 study. Following analyses were proceeded to: (i) collect genes with TPM > 0.1 in at least 20% of samples and median TPM > 0.5 in AA and LV, (ii) identify heart-specific/enhanced genes including protein-coding genes and DEGs, (iii) test enrichment of the top 500 genes having high relative median values and heart-specific/enhanced genes, (iv) perform text-mining of PubMed abstracts, and (v) conduct Ingenuity Pathway Analysis (IPA) for three groups (heart-specific/enhanced genes, DEGs, and unreported genes).

**FIGURE 2:**
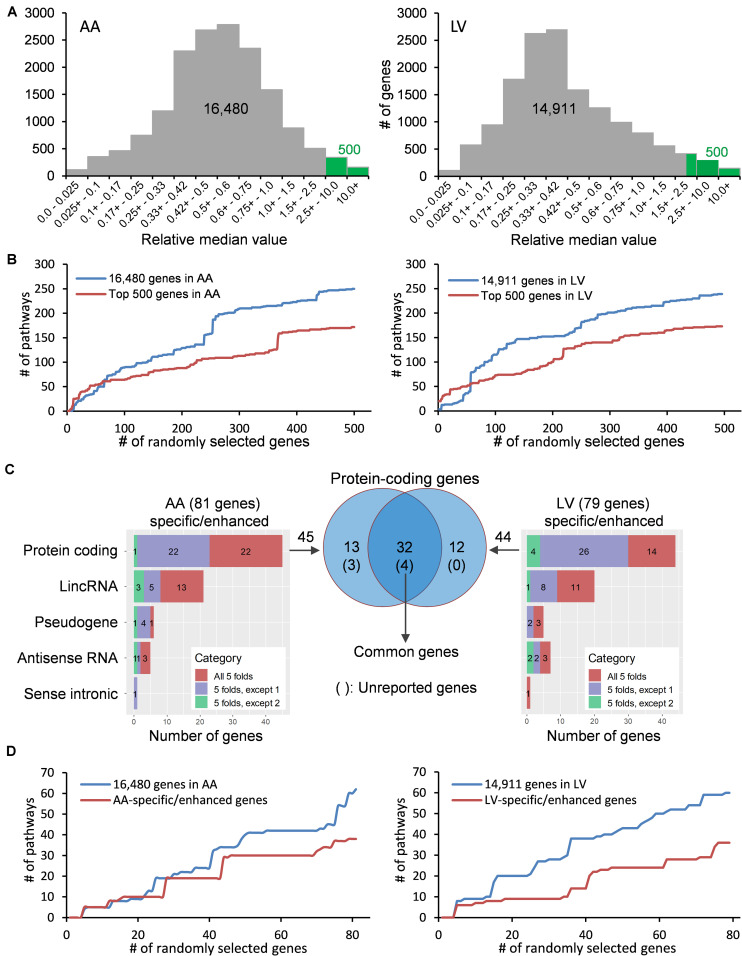
Distribution and classification of genes highly expressed in the heart. **(A)** Median TPMs in AA and LV are divided by an average of other tissues’ median TPMs, and the resulting relative median values (RMVs) are distributed. Green bars represent the top 500 genes with relative median values of more than 2.51-fold and 2.32-fold in AA and LV, respectively. **(B)** The number of enriched KEGG pathways were plotted against cumulatively increased randomly selected genes from two groups (all genes passed cutoffs and the top 500 genes) in AA and LV. **(C)** Types and numbers of heart-specific genes whose median expression is more than 5-fold higher in the heart compared to all other tissues (All 5-folds), and heart-enhanced genes whose median expression is more than 5-fold higher in heart tissue compared to all other tissues, except one tissue (5-folds, except 1) and two tissues (5-folds, except 2). The significance of differences under ANOVA-based test was set at a threshold of FDR < 0.01. Venn diagram shows genes whose expression is specific or enhanced in both AA and LV as indicated in the intersection (*n* = 32), among which four genes are unreported in PubMed regarding the heart. **(D)** Cumulative enrichment test for AA- and LV-specific genes with random selection.

Comprehensive analyses of human transcriptome across tissues were initiated by downloading on 02/22/2019 public human RNA-seq data (GTEx Analysis v7; dbGaP Accession: phs000424.v7.p2, release date: June 30, 2017) deposited in the GTEx portal^2^) (Figure 1). Gene expression values in the data were derived from non-diseased normal tissues of postmortem human donors (aged 21–70) and normalized using transcripts per million (TPM). Before processing the data, tissues from less than 30 donors and non-tissue samples such as cultured transformed fibroblasts and EBV-transformed lymphocytes were excluded from the current study. Then, tissue samples with high quality RNA (RNA integrity number, RIN, of 6.0 or higher) were selected. Finally, the filtered data included 47 human tissues, and the sample size (i.e., donor numbers) for each tissue ranged from 35 to 560 (Supplementary Table S1). In addition, 56,202 human protein-coding and non-coding genes were filtered by general thresholds of at least 20% of samples having TPM > 0.1^3^) and median TPM > 0.5 in the heart (Ahn et al., 2019), leaving 16,480 and 14,911 protein-coding and non-coding genes for data analysis for the atrial appendage (AA) and left ventricle (LV), respectively, (Figure 2A).

### Cumulative Pathway Enrichment

Enrichment analysis on randomly selected genes was conducted as follows. From the above 16,480 (AA) and 14,911 (LV) genes, the top 500 genes were selected by the rank of relative median value. From either 16,480 (or 14,911) genes or the top 500 genes, 5 to 500 genes were randomly selected by adding one gene per new selection, using the sample_n function of the dplyr package (v0.8.5^4^). The same random selection procedure was also applied for AA-specific/enhanced genes (5 to 81 genes) and LV-specific/enhanced genes (5 to 79 genes). The kyoto Encyclopedia of Genes and Genomes (KEGG) pathway was selected as a reference database, and we ran an Enrichr algorithm^5^ (Chen et al., 2013; Kuleshov et al., 2016) to query to the KEGG 2019 human database (308 terms, gene coverage of 7,802, and 92 genes per term) as presented in a previous publication (Zachariou et al., 2018). In the current study, a Python script (enrichr-cli.py, W. De Coster^6^) was used to run the algorithm and obtain Enrichr combined scores for input gene lists, which are derived from a correction to the Fisher’s exact test *p*-value by the *z*-score of deviation from the expected rank (Chen et al., 2013).

### Data Processing and Statistical Analysis

Heart-specific genes were defined as genes with fold changes of the median expression levels (FCMs) higher than 5.0 in the heart *versus* all other tissues. Heart-enhanced genes were defined as genes having FCMs higher than 5.0 in the heart *versus* all other tissues, with an exception of 1 or 2 other tissues, and a higher median expression in the heart in the case of exceptions. That is, we selected a trend in which the heart showed a greater expression than any other analyzed tissues even in the case of heart-enhanced genes as well as heart-specific genes.

Using lmFit function in the R/Bioconductor limma package (Ritchie et al., 2015), a linear model was fitted to our data and a contrast matrix was created for all 45 pair-wise comparisons between each tissue. In limma, the empirical Bayes (eBayes) method (Smyth, 2004) followed by the decideTests function with “global” option was used to test *t*-statistic for each contrast and F-statistics for all 45 contrasts simultaneously, along with multiple testing correction by the Benjamini–Hochberg’s procedure (Benjamini and Hochberg, 1995). As a result, genes with at least a 5-fold higher median expression in the heart and whose expression is significantly different between tissues, formulated as: [FDR of *i versus j* pairwise comparison] < 0.01, where *i* = A1 (heart-atrial appendage) or A2 (heart-left ventricle) and *j* = B, C, D, ⋯, or AT (non-heart), were selected.

For grouping protein-coding and non-coding genes (81 genes in AA and 79 genes in LV) related to long intergenic non-coding RNA (linc RNA), pseudogene, antisense RNA, and sense intronic transcripts, the Ensembl Gene IDs retrieved from the GTEx portal (hg19/GRCh37) were reannotated using the human reference genome assembly (GRCh38.p12^7^). Heat maps were generated to visualize relative heart-specific and heart-enhanced expressions using the R package “heatmap3”^8^ (Zhao et al., 2014).

### Literature Mining

To access to PubMed^9^ and download abstracts of publications on the heart and heart-related medical conditions, the PubMed literature database was queried on May 26, 2020 using the R package “easyPubMed”^10^. Briefly, the PubMed database was queried without restriction on publication date and using search terms independently for each of the 57 heart- specific/enhanced protein-coding genes as follows: ‘heart[TIAB] OR atrial[TIAB] OR ventricle[TIAB] OR cardio[TIAB] OR cardiac[TIAB] OR coronary[TIAB] OR cardium[TIAB] AND (genesymbol [TIAB])’, where TIAB stands for title/abstract and genesymbol was replaced with each different gene symbol. Abstracts were downloaded in XML format. Extraction of biomedical entities such as genes, diseases, species, and PubMed identifier (PMID) from the downloaded abstracts was performed using the R package “pubmed.mineR”^11^ (Rani et al., 2015). When no abstract was retrieved for a certain gene by easyPubMed, it was referred to as a previously “unreported gene” in PubMed (hereafter unreported gene). When an abstract was retrieved and biomedical entities were extracted, it was referred to as a “reported gene” in PubMed (hereafter, a reported gene). For some cases in which abstracts were retrieved but related diseases were not extracted, a manual review on abstracts and full texts under each extracted PMID was performed as a part of data mining strategy (Pospisil et al., 2006) (e.g., research on non-pathological conditions such as heart rate was regarded as a reported case).

### Ingenuity Pathway Analysis

The Ingenuity Pathway Analysis (IPA) was used to perform pathway enrichment analysis as follows. IPA Comparison Analysis was conducted on two groups of 20 tissues (i.e., either AA and 19 other tissues or LV and 19 other tissues) to examine similarities and differences in pathway enrichment. The selection of 20 tissues was due to the maximum analyzing capacity of IPA Comparison Analysis of 20 observations (or experimental groups), and tissues with greater size and larger sample numbers were chosen. Among the top 500 genes, all heart-specific/enhanced genes were analyzed, and their log ratios (i.e., log_2_RMVs) were used to rank them. For those heart-specific/enhanced genes, adjusted *p*-values of differential expression between tissue *i versus rest*, where *i* is one tissue from 20 tissues and *rest* is 19 other tissues, were obtained from the limma package using a contrast matrix of one *versus* 19 followed by the eBayes method and t-statistic. Using log_2_RMVs and adjusted *p*-values, IPA Core Analysis was performed on each of the 20 tissues and then IPA Comparison Analysis was conducted using 20 Core Analysis results. Enrichment was considered significant when *p* < 0.01. A cutoff set for activation or inhibition of the pathways predicted by IPA was |activation *z*-score| ≥ 2.

### Differential Expression Between Atrial Appendage and Left Ventricle

Read count matrix from RNA-seq of atrial appendage and left ventricle was downloaded from the GTEx portal on 02/22/2019. Read counts ≥ 6 in at least 20% of samples^12^ was satisfied with 23,288 genes in AA and 22,137 genes in LV, and the union (23,610 genes) of the two gene sets were retained. For differential expression analysis, the R/Bioconductor “DESeq2” package (Love et al., 2014) was used to normalize samples for sequencing depth and calculate DEGs between AA and LV. To obtain significant DEGs, combined criteria of FDR < 0.01 and absolute value of log_2_(fold change) > 3 were used, where a fold change is defined as the expression in samples of AA divided by that of LV. IPA Core Analysis was conducted to analyze pathway enrichment of those significant DEGs, using log_2_(fold change) and adjusted *p-*values as input values. The *p* < 0.01 was considered a significant enrichment. The |activation *z*-score| ≥ 2 was used to predict activation or inhibition of the pathways.

## Results

### Data Distribution and Cumulative Pathway Enrichment

With the purpose of identifying heart-specific genes and their associations with diseases and traits, our integrative workflow was designed (Figure 1). To initiate the workflow, the human RNA-seq-based transcriptome data (GTEx Analysis v7) were downloaded from the GTEx portal^13^. In each tissue, the medians of gene expression values (TPMs) were obtained in virtue of their robustness to outliers. Then, the median TPM value in the heart was divided by an average of the rest of the median TPMs from other tissues, producing ‘relative median values (RMVs)’, and distribution of these values were plotted against the number of genes (Figure 2A). The plots were relatively symmetric and displayed about one hundred and fifty values with more than 10-fold, indicating the presence of exclusive or abundant expression in the two heart tissues: atrial appendage and left ventricle.

To examine whether the degree of enrichment changes by selecting genes with high RMVs, the number of enriched pathways were queried by increasing the input gene set cumulatively (Figure 2B). At first, five genes were randomly selected from two groups: one is the gene set with all valid genes (i.e., pre-selected 16,480 genes for AA or 14,911 genes for LV) and another is with the top-ranked 500 genes (more than 2.51- and 2.32-fold in AA and LV, respectively). For the cumulative increase, one gene was added into the gene set each time after random selection from either group. The KEGG 2019 human database was selected as a reference pathway database for this test query owing to its comprehensiveness and a wide range of usage (Kanehisa et al., 2017). Upon running through the Enrichr algorithm, cumulative numbers of total enriched pathways with Enrichr combined scores, which outperform other measurements on enrichment (Kuleshov et al., 2016; Zachariou et al., 2018), were plotted for each gene list with respect to numbers of randomly selected genes (Figure 2B).

As shown in Figure 2B, in either case, with less than about 50 to 70 genes, the number of total enriched pathways was higher in gene sets selected from the top 500 genes compared to the all genes. However, for larger gene sets, the number of enriched pathways for the top 500 gene set became substantially smaller than the all genes, and this pattern was continued in both cases of AA and LV. It indicated that the extent of enrichment was affected by RMVs and choosing genes with high RMVs increased the detection sensitivity. Taken together, highly expressed genes in the heart could be detected by sorting RMVs, and selection of the top 500 genes improved enrichment results.

### Identification of Heart-Specific and Heart-Enhanced Genes

In order to identify more specific genes in the heart, we further narrowed down those top 500 genes to “heart-specific genes” and “heart-enhanced genes” using rigorous criteria as described in the section “Materials and Methods.” As a result, we selected a total of 81 protein coding and non-coding genes in AA and 79 in LV (all FCMs > 5 with up to 2 exceptions, FDR < 0.01; Figure 2C and Supplementary Table S1). Among them, the number of protein coding genes were 45 and 44 in AA and LV, respectively, and there were 32 overlapping genes. In addition, with the same random selection strategy used in the case of the top 500 genes (Figure 2B), those AA- and LV-specific/enhanced genes were subjected to the cumulative pathway enrichment test and showed a more refined enrichment than all genes similar to the top 500 genes (Figure 2D). It also indicated that the detection sensitivity increased with AA- and LV-specific/enhanced genes because the refined enrichment occurred with smaller gene sets, compared to the top 500 genes, and these specific/enhanced genes were further studied below.

**FIGURE 3:**
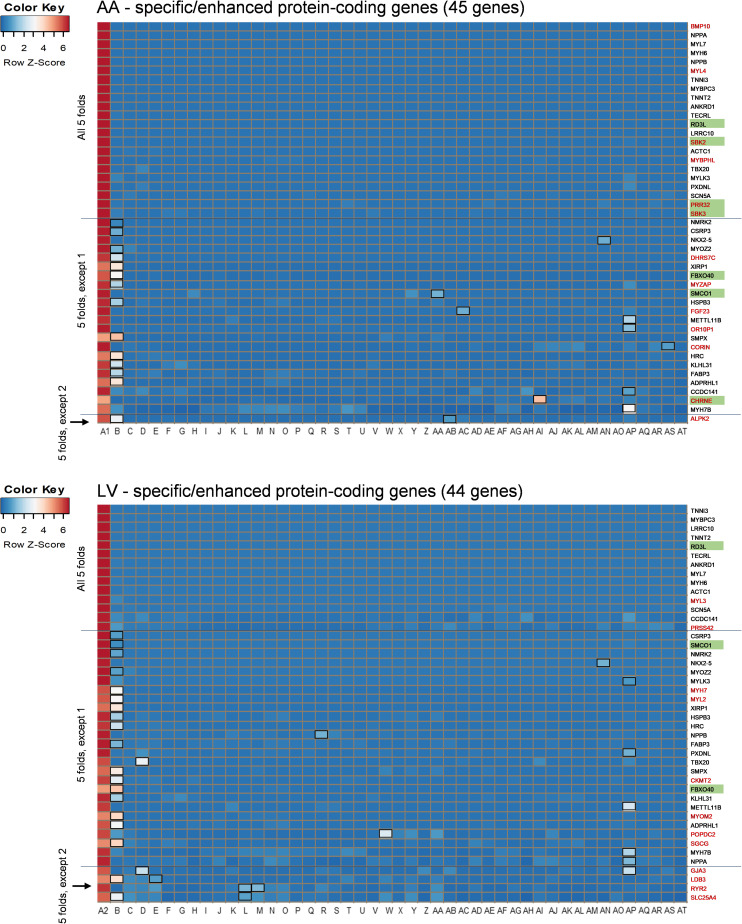
Heat maps of heart-specific and heart-enhanced genes. Visualization is based on relative median expression levels of heart-specific (all median 5-folds) and heart-enhanced (median 5-folds, except 1 & median 5-folds, except 2) genes in 47 human tissues [A1: Heart – Atrial Appendage, A2: Heart – Left Ventricle, and B through AT: 45 non-heart tissues (B: Muscle – Skeletal, C: Artery – Aorta, D: Artery – Coronary, E: Artery – Tibial, F: Adipose – Subcutaneous, G: Adipose – Visceral (Omentum), H: Adrenal Gland, I: Brain – Amygdala, J: Brain – Anterior cingulate cortex (BA24), K: Brain – Caudate (basal ganglia), L: Brain – Cerebellar Hemisphere, M: Brain – Cerebellum, N: Brain – Cortex, O: Brain – Frontal Cortex (BA9), P: Brain – Hippocampus, Q: Brain – Hypothalamus, R: Brain – Nucleus accumbens (basal ganglia), S: Brain – Putamen (basal ganglia), T: Brain – Spinal cord (cervical c-1), U: Brain – Substantia nigra, V: Breast – Mammary Tissue, W: Colon – Sigmoid, X: Colon – Transverse, Y: Esophagus – Gastroesophageal Junction, Z: Esophagus – Mucosa, AA: Esophagus – Muscularis, AB: Kidney – Cortex, AC: Liver, AD: Lung, AE: Minor Salivary Gland, AF: Nerve – Tibial, AG: Ovary, AH: Pancreas, AI: Pituitary, AJ: Prostate, AK: Skin – Not Sun Exposed (Suprapubic), AL: Skin – Sun Exposed (Lower leg), AM: Small Intestine – Terminal Ileum, AN: Spleen, AO: Stomach, AP: Testis, AQ: Thyroid, AR: Uterus, AS: Vagina, AT: Whole Blood)]. Genes specific or enhanced in either atrial appendage or left ventricle are shown in red letters, and common genes overlapping between atrial appendage and left ventricle are denoted by black letters. Functionally unreported heart-specific/enhanced genes are indicated with green filled rectangles. Tissues with less than a 5-fold difference in medians are marked with black unfilled rectangles in the heat maps. *Z*-scores of RMVs were calculated by heatmap3 for each row (i.e., each gene). By selecting the heart-specific/enhanced genes, heat maps were skewed to positive *z*-scores compared to Supplementary Figures S2, S3. |*z*-score| ≥ 2 indicates at least 2 standard deviation from the mean.

Among those overlapping genes, three genes (*RD3L*, *FBXO40*, and *SMCO1*) have not been documented in PubMed (i.e., unreported genes) regarding their related diseases and functions in the heart in humans based on our literature search. Genes whose function has been investigated in non-pathological conditions such as *CCDC141* on heart rate (van de Vegte et al., 2019), as well as disease conditions, were classified as reported genes. Among non-overlapping genes, 13 protein-coding genes were AA-specific/enhanced and among them the following four genes were unreported genes: *SBK2*, *PRR32*, and *SBK3* showed exclusive expression (AA-specific), and expression of *CHRNE* was more than 5-fold compared to other tissues, except pituitary (AA-enhanced). In addition, 11 non-overlapping LV-specific/enhanced protein coding genes were found and all of them were reported. Heat maps displayed *z*-scores of RMVs of the heart-specific/enhanced protein-coding genes in various tissues (i.e., each row) with extreme specificity or abundancy to AA and LV (Figure 3). Those heat maps were extended for 16,480 genes in AA and 14,911 genes in LV and divided by each chromosome, displaying specificity or abundancy of the heart-specific/enhanced protein-coding and non-coding genes in each chromosome compared to the rest of the genes (Supplementary Figures S1, S2).

### Identification of Enriched Diseases and Biological Functions

**FIGURE 4:**
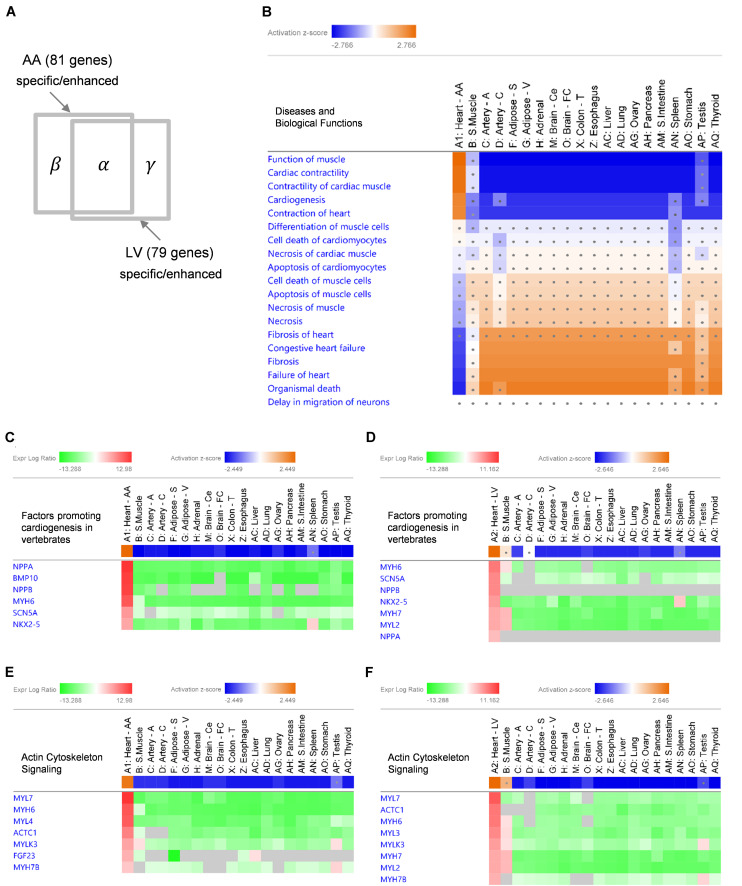
IPA Comparison Analysis of specific/enhanced protein-coding and non-coding genes in 20 tissues. **(A)** A diagram showing an intersection of 53 common-specific/enhanced genes (α), 81 AA-specific/enhanced genes (α + β), and 79 LV-specific/enhanced genes (α + γ). **(B)** A heat map of enriched ‘Diseases and Biological Functions’ with common-specific/enhanced genes (α). For each tissue, log_2_RMV and FDR of each gene compared to other tissues were calculated by the limma package as an input for the analysis. Enriched IPA terms out of ∼900 ‘Diseases and Biological Functions’ terms are listed. Activation *z*-scores calculated by IPA software indicate prediction of activation (orange) or inhibition (blue) of each pathway in each tissue. Ranges of color keys were automatically selected by IPA software, and a significant activation *z*-score was set to greater than or equal to 2 standard deviation (i.e., |*z*-score| ≥ 2) and dots denote non-significant *z*-scores (i.e., | *z*-score| < 2). **(C–F)** Gene Heatmaps for Canonical Pathways of ‘Factors Promoting Cardiogenesis in Vertebrates’ **(C,D)** and ‘Actin Cytoskeleton Signaling’ **(E,F)**. Activation *z*-scores of canonical pathways and expression log ratios (i.e., log_2_RMV) of AA-specific/enhanced genes (α + β) **(C,E)** and LV-specific/enhanced genes (α + γ) **(D,F)** are displayed. Overall, symbols for each tissue (A1 or A2 through AQ) are consistent with Figure 3.

Given the extreme specificity and abundancy of the heart-specific/enhanced genes, pathological or functional implications of those genes were explored. Using IPA software, enriched “Diseases and Biological Functions” were identified in the following three groups of gene sets: 58 common-specific/enhanced protein-coding and non-coding genes (α), 81 AA-specific/enhanced protein-coding and non-coding genes (α + β), and 79 LV- specific/enhanced protein-coding and non-coding genes (α + γ) (Figure 4A). Based on the aforementioned cumulative enrichment test, more than 50 specific/enhanced genes were selected to produce enrichment results with IPA.

With common-specific/enhanced genes (α), out of a total of approximately 900 terms listed in “Diseases and Biological Functions,” 18 terms had *z*-scores in at least one tissue among 20 tissues. Among them, 5 functional terms (Function of Muscle, Cardiac Contractility, Contractility of Cardiac muscle, Cardiogenesis, and Contraction of Heart) were predicted to be activated (*z*-score ≥ 2) and 4 disease terms (Congestive Heart Failure, Fibrosis, Failure of Heart, and Organismal Death) were predicted to be inhibited (*z*-score ≤ −2), both when common-specific/enhanced genes were compared between AA and other tissues (Figure 4B) and between LV and other tissues (Supplementary Figures S3A,B). Most other tissues showed opposite prediction of activation or inhibition, and prediction was not significant for skeletal muscle (for all of the above 9 terms), testis (8 terms), spleen (5 terms) and coronary artery (2 terms) showing these diseases and biological functions were not oppositely inhibited or activated in those tissues (Figure 4B and Supplementary Figure S3B). Nonetheless, none of the other tissues showed the same pattern of prediction as the heart, indicating the exclusive role of common-specific/enhanced genes in promoting those biological functions and inhibiting those diseases.

With AA-specific/enhanced genes (α + β), 6 terms were predicted to be activated (*z*-score ≥ 2) and four terms were predicted to be inhibited (*z*-score ≤ −2) in AA (Supplementary Figure S3C), and with LV-specific/enhanced genes (α + γ), 7 terms were predicted to be activated (*z*-score ≥ 2) and 5 terms were predicted to be inhibited (*z*-score ≤ −2) in LV (Supplementary Figure S3D). Those terms were all related to activation in cardiac muscle contraction and cardiogenesis (functional terms) and inhibition of heart failure, fibrosis and organismal death (disease terms), as shown in the case of common-specific/enhanced genes. It suggested essential roles of common-specific/enhanced genes in promoting contraction of the heart and cardiac development and preventing dysfunction and failure of the heart.

### Characteristics in Enriched Canonical Pathways

Specific canonical pathways were further explored to compare common-specific/enhanced genes with AA- or LV-specific/enhanced genes. From IPA, prediction of activation for “Factors Promoting Cardiogenesis in Vertebrates” were found to be higher in AA (*z*-score 2.45) and LV (*z*-score 2.65), compared to common-specific genes (*z*-score 2.24). The AA- or LV-specific/enhanced genes, that are not common-specific/enhanced and might increase those *z*-scores were *BMP10* in AA and *MYH7* (or β*-MHC*) and *MYL2* (or *MLC2v*) in LV (Figures 4C,D). In this study, the *BMP10* gene showed the highest specificity (Figure 3) but the expression ratio was slightly changed when comparing 20 tissues for IPA ranking *BMP10* the second in Figure 4C. The *MYH7* and *MYL2* genes were LV-enhanced genes (Figure 3).

In addition, in relation to cardiac muscle contraction, prediction of activation for another canonical pathway “Actin Cytoskeleton Signaling” was higher in AA (*z*-score 2.24) and LV (*z*-score 2.65), compared to common-specific genes (*z*-score 2.00). The AA- or LV-specific/enhanced genes, that are not common-specific/enhanced and might increase those *z*-scores were *FGF23* and *MYL4* in AA and *MYL3*, *MYH7* and *MYL2* in LV (Figures 4E,F).

For disease terms, prediction of inhibition was increased the most for “Organismal Death” with AA-specific/enhanced genes (*z*-score −3.05) and LV-specific/enhanced genes (*z*-score −3.07) compared to common-specific/enhanced genes (*z*-score −2.38). Also, prediction of inhibition for “Failure of Heart” (*z*-score −2.219) with common-specific/enhanced genes was increased with AA-specific/enhanced genes (*z*-score −2.43), and “Fibrosis” (*z*-score −2.09) with common-specific/enhanced genes was predicted to be inhibited more with LV-specific/enhanced genes (*z*-score −2.31). It suggests that those above canonical pathways related to cardiogenesis and cardiac muscle contraction and AA- and LV-specific/enhanced genes might be related to suppressing these pathological conditions and promoting organismal survival and function.

### Atrial Appendage-Related BMP and FGF Signaling and Differences in Left Ventricle

**FIGURE 5:**
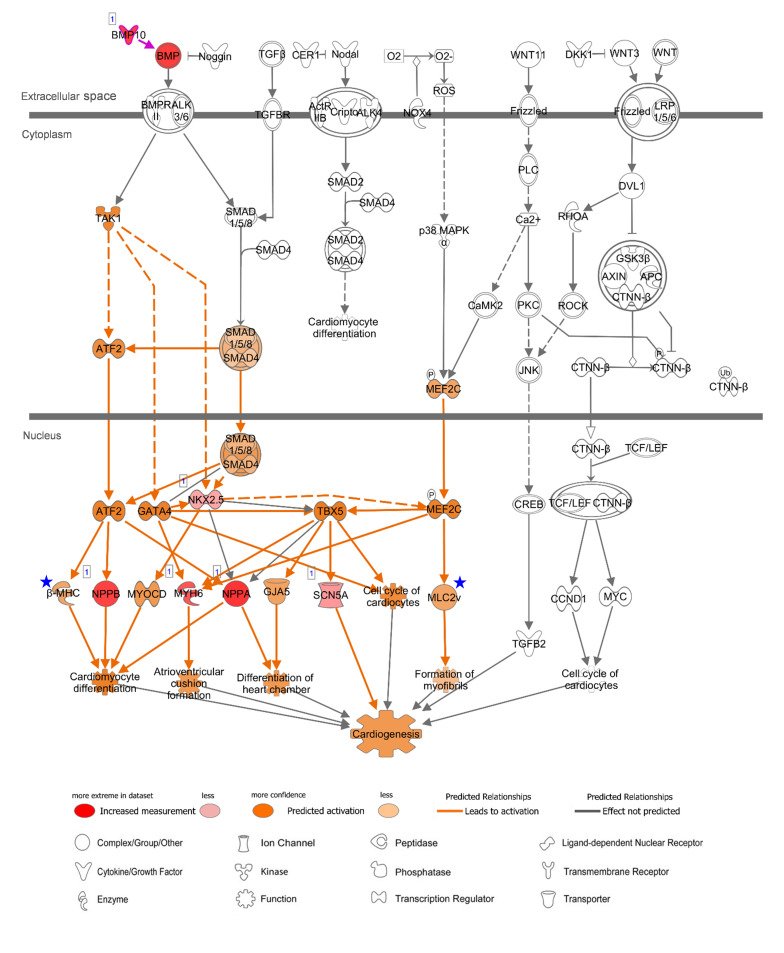
Canonical Pathway of ‘Promoting Cardiogenesis in Vertebrates’ in IPA with AA-specific/enhanced genes in Figure 4C. Up-regulated expression in AA was indicated with *red nodes*. Prediction of activation for molecules (*nodes*) and for relations (*lines*) are colored with *orange*. The color intensity represents the relative magnitude of changes in gene expression or prediction of activation. Direct and indirect relations are indicated by *solid* and *dashed lines*, respectively. White molecules represent their absence in the input gene set. *Gray lines* indicate that direction of change was not predicted. *Number one* on the top left of the genes indicate a single form. *Blue stars* indicate LV-enhanced genes, *MYH7* and *MYL2*, in Figure 4D, that were up-regulated in the case of LV regarding this canonical pathway. Data were analyzed through IPA to generate the canonical pathway (https://www.qiagenbioinformatics.com/products/ingenuity-pathway-analysis).

In order to gain functional and mechanistic insights, the above canonical pathways were further investigated. In relation to “Factors Promoting Cardiogenesis in Vertebrates,” bone morphogenetic protein 10 (BMP10) is a peptide growth factor and both SMAD-dependent and independent pathways were predicted to be activated via phosphorylation (Figure 5). A subsequent binding of SMAD1/5/8 and co-SMAD (SMAD4) and activation of *NKX2.5*, the determinant of cardiac development and the common-enhanced gene in our case, through both SMAD complex and GATA-4 (Brown et al., 2004) might be a key determination step of cardiogenesis in atrial appendage. The role of *BMP10* in the adult heart remains elusive, but as reported recently *BMP10* may play multifunctional roles not only in promoting cardiac development in prenatal stages but also in inhibiting cardiomyocyte apoptosis and cardiac fibrosis in adults (Qu et al., 2019). In the downstream of this canonical pathway, cardiac transcription factors were predicted to activate target genes including AA-specific and LV-enhanced genes: natriuretic peptide B (*NPPB*) and natriuretic peptide A (*NPPA*). These paralogous genes are transcriptionally regulated as a cluster and encode peptide hormones that are secreted by cardiomyocytes (Man et al., 2018), and was predicted to induce cardiomyocyte differentiation (Figure 5). The expression of the other two target genes, sodium voltage-gated channel alpha subunit 5 (*SCN5A*) that encodes an integral membrane protein, a sodium channel, for transmitting electric signals to maintain normal heart contraction (Zaklyazminskaya and Dzemeshkevich, 2016) and myosin heavy chain 6 (*MYH6*) which encodes the sarcomeric filament protein alpha-myosin heavy chain (Posch et al., 2011), were relatively lower than *NPPB* and *NPPA*. In LV, additional genes include myosin heavy chain 7 (*MYH7* or β*-MHC*) that encodes beta-myosin heavy chain and myosin light chain 2 (*MYL2* or *MLC2v*) that encodes regulatory myosin light chain that are up-regulated as LV-enhanced genes.

**FIGURE 6:**
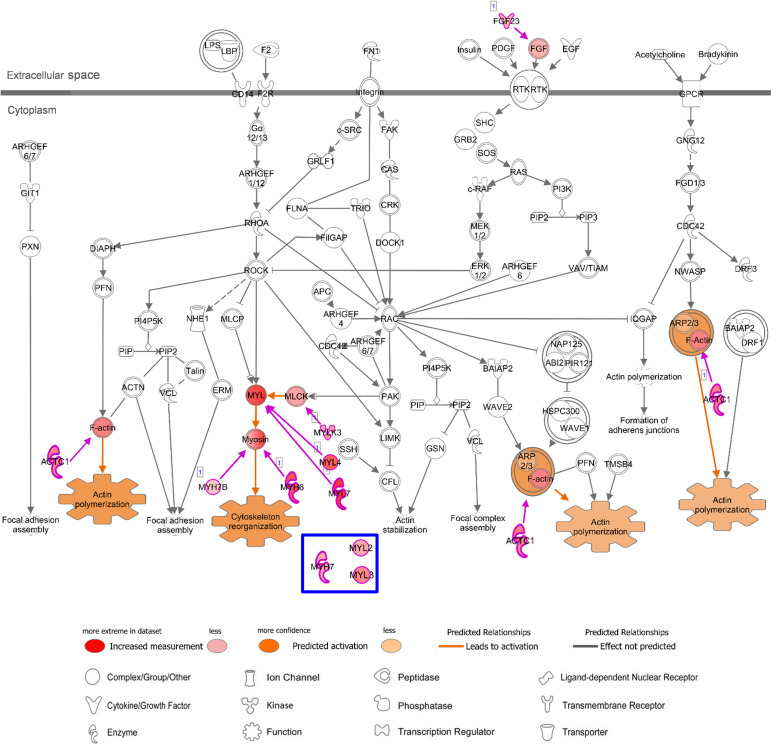
Canonical Pathway of ‘Actin Cytoskeleton Signaling’ in IPA with AA-specific/enhanced genes in Figure 4E. Details of figure legends are in Figure 5. Molecules in *blue box* indicate a LV-specific gene (*MYL3*) and LV-enhanced genes (*MYH7* and *MYL2*) in Figure 4F, that were up-regulated with this canonical pathway in the case of LV.

In addition, regarding “Actin Cytoskeleton Signaling” which was predicted to be activated with heart-specific/enhanced genes, one of the genes that were additionally involved in this enriched pathway in AA was fibroblast growth factor 23 (*FGF23*), which is an AA-enhanced gene (Figure 6). Fibroblast growth factor 23 (FGF23) is a circulating hormone that is associated with cardiac hypertrophy and also acutely elevates intracellular Ca^2+^ and increases cardiac contractility (Touchberry et al., 2013). Binding of FGF23 to receptor tyrosine kinases leads to tyrosine phosphorylation and stimulation of Ras/MARK and PI3K/Akt signaling (Grabner et al., 2015). Another additional gene in AA was myosin light chain 4 (*MYL4*), a previously known atrial-specific myosin light chain, whose mutation causes familial atrial fibrillation (Orr et al., 2016). In LV, *MYH7*, *MYL2*, and myosin light chain 3 (*MYL3*) were additionally involved in this canonical pathway. Although both AA-specific/enhanced genes and LV-specific/enhanced genes were predicted to promote actin polymerization and cytoskeleton reorganization, those different cardiac sarcomere protein genes might give rise to distinct contractile and electrophysiological properties between AA and LV.

### Differential Expression Between Atrial Appendage and Left Ventricle

**FIGURE 7:**
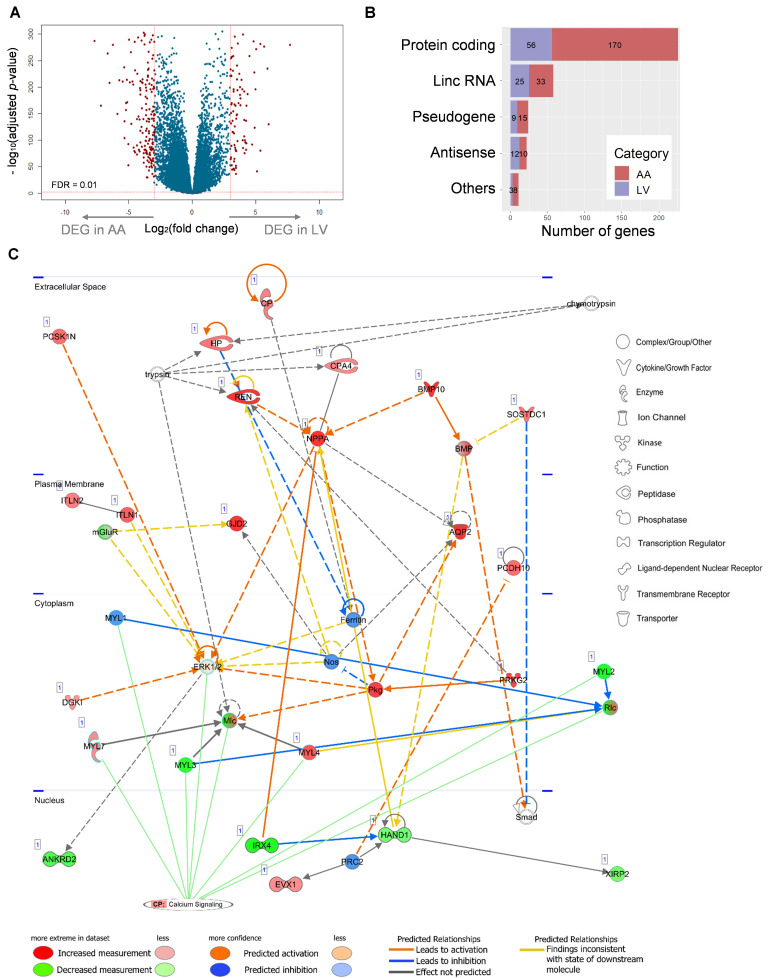
Relationship between a network of DEGs and the canonical calcium signaling pathway. **(A)** Differential expression analysis of 23,610 genes and volcano plot representation of DEGs. The negative log10-transformed adjusted *p*-value was plotted against the log ratios (fold change) between AA and LV. Red dots represent 236 and 105 DEGs in AA and LV, respectively, that show both at least 8-fold changes and high statistical significance (FDR < 0.01). **(B)** DEGs were categorized into five groups including protein-coding, lincRNA, pseudogene, antisense RNA, and others (processed transcript and sense intronic). Numbers of DEGs in each category are marked on the bar graph. **(C)** The top scoring IPA network related to DEGs: ‘Cardiovascular System Development and Function, Embryonic development, Organ development’. Up- (*red*) and down- (g*reen*) regulated genes (*nodes*) are indicated. *Orange and blue lines* indicate prediction of activation and inhibition, respectively, while *gray lines* indicate no prediction for direction of changes. *Solid* and *dashed lines* represent direct and indirect relations, respectively. Calcium signaling is shown as a pathway that has the highest prediction of activation. *CP*, canonical pathway. The networks were generated through IPA (QIAGEN Inc., https://www.qiagenbioinformatics.com/products/ingenuity-pathway-analysis).

A total of 341 DEGs (Log_2_(fold change) ≥ 3, FDR < 0.01) were identified, among which 236 and 105 DEGs showed up-regulated expression in AA and LV, respectively, (Figure 7A and Supplementary Table S2). Protein-coding DEGs were 170 and 55 in atrial appendage and left ventricle, respectively, (Figure 7B). By doing IPA Core Analysis on 341 DEGs, twenty networks regarding diseases and functions were identified. Among them, the top ten networks were scored 19 to 42 [*p*-score = −log_10_(*p*-value)] with 13 to 23 focus molecules (Supplementary Table S3). The first-ranked network was “Cardiovasuclar System Development and Function, Embryonic Development, Organ Development” with 23 focus molecules (*p*-score = 42). In this network, a subnetwork with the most significant enrichment was “Morphogenesis of Cardiac Muscle” (*p* = 8.96 × 10*^–^*^7^) with 4 molecules: *BMP10*, *HAND1*, *MYL3*, and *XIRP2*. Among those four genes, *BMP10* was upregulated in AA (log_2_FC = 12.38), and the other three genes were downregulated in AA (*HAND1* log_2_FC = −3.03, *MYL3* log_2_FC = −4.50, and *XIRP2* log_2_FC = −3.05) and upregulated in LV. Another significant subnetwork was “Hypertrophy of Left Ventricle” (*p* = 4.08 × 10*^–^*^5^), and related molecules were *IRX4* (log_2_FC = −7.33), *MYL3* (log_2_FC = −4.50), *NPPA* (log_2_FC = 6.05), and *REN* (log_2_FC = 7.20).

The network between 23 focus molecules under the first-ranked “Cardiovasuclar System Development and Function, Embryonic Development, Organ Development,” including the top two subnetwork molecules (the above seven molecules), was displayed (Figure 7C). In addition, the relationship of this network to canonical pathways was examined and the “Calcium Signaling” pathway showed a significant enrichment (*p* = 3.31 × 10*^–^*^5^) and the highest prediction of activation (*z*-score = 2.24) with this experimental group (the combination of up-regulated DEGs in AA and down-regulated DEGs in LV) (Figure 7C). Eight molecules (*MYL7*, *MYL1*, *MYL3*, *ERK1/2*, *Mlc*, *MYL4*, *MYL2*, and *Rlc*) were involved in this calcium signaling pathway. On the other hand, other canonical pathways that were related to AA- and LV-specific/enhanced genes were not significantly enriched or not predicted to be activated or inhibited: “Factors Promoting Cardiogenesis in Vertebrates” (*p* = 0.06, *z*-score = 0) and “Actin cytoskeleton signaling” (*p* = 5.37 × 10*^–^*^3^, *z*-score = −0.447). It suggests that there may be functional differences between heart-specific/enhanced genes and DEGs.

### Novel Insights on Unreported Heart-Specific/Enhanced DEGs

In order to find heart-specific/enhanced genes that are differentially expressed between AA and LV and are previously unreported in PubMed, we first compared heart-specific/enhanced genes with DEGs. We found that 9 AA-specific/enhanced protein-coding genes were up-regulated DEGs in AA. Thus, these genes (*BMP10*, *MYBPHL*, *CHRNE*, *NPPA*, *SBK2*, *MYL4*, *OR10P1*, *PRR32*, and *MYL7*) were “AA-specific/enhanced DEGs” meaning that they were specific/enhanced to AA compared to 45 non-heart tissues as well as up-regulated in AA compared to LV (Supplementary Table S2). Also, there were 3 LV-specific/enhanced protein-coding genes that were up-regulated DEGs in LV (*MYL2*, *MYL3*, and *MYH7*), and they were named ‘LV-specific/enhanced DEGs’ which were specific/enhanced to LV compared to 45 non-heart tissues as well as up-regulated in LV compared to AA.

**FIGURE 8:**
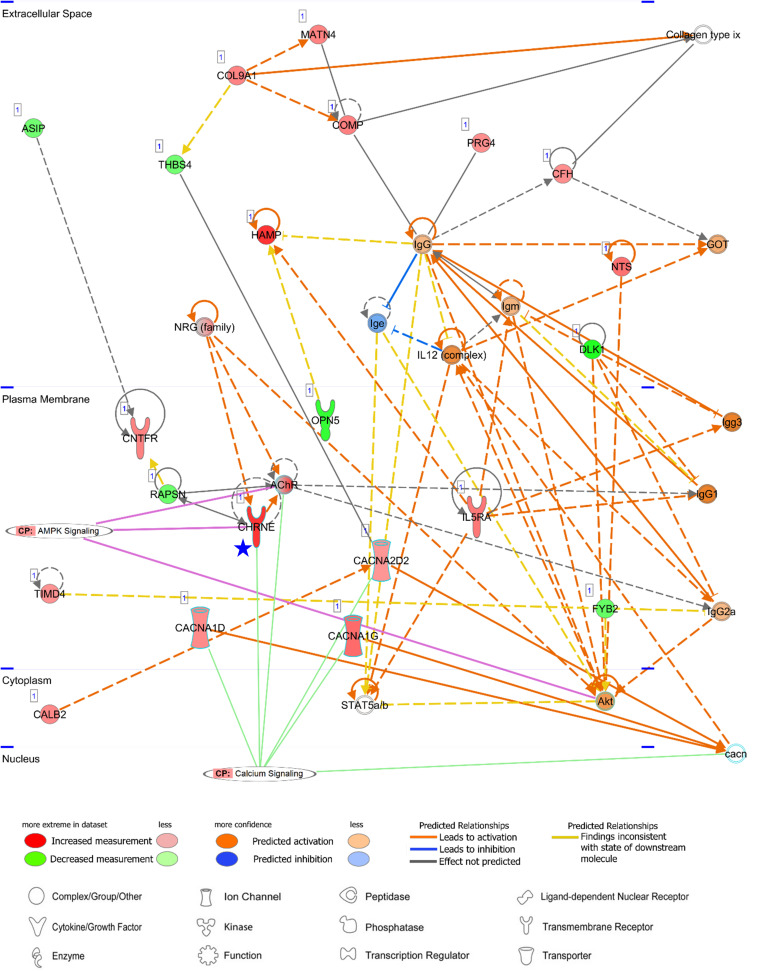
Networks of an unreported AA-specific/enhanced DEG (*CHRNE*). AMPK signaling and calcium signaling are shown as related pathways in the networks. *CP*, canonical pathway. A *Blue star* pointed *CHRNE* which is unreported regarding the heart. Details regarding the network in Figure 7C.

**FIGURE 9:**
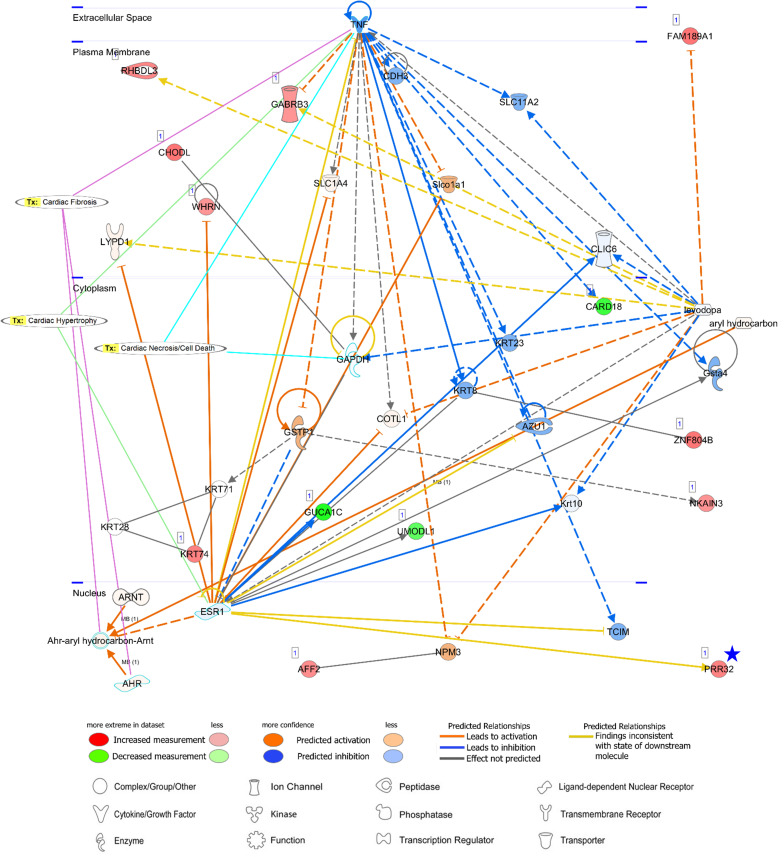
A network analysis for an unreported AA-specific/enhanced DEG (*PRR32*) indicated with a *blue star*. Relationships between genes (*nodes*) and heart diseases (cardiac fibrosis, cardiac hypertrophy, and cardiac necrosis/cell death) are indicated. *Tx*, toxicity-related lists. Details on the network are in Figure 7C.

Among both AA- and LV-specific/enhanced DEGs, three AA-specific/enhanced DEGs [cholinergic receptor nicotinic epsilon subunit (*CHRNE*), SH3 domain binding kinase family member 2 (*SBK2*), and proline rich 32 (*PRR32*)] were “unreported” genes in PubMed regarding the heart. On the other hand, all the LV-specific/enhanced DEGs are known myosin genes. From the IPA Network Analysis for DEGs, networks for two out of the three AA-specific/enhanced DEGs were found (Supplementary Table S3). The *CHRNE* gene was one of the focus molecules under “Developmental Disorder, Hereditary Disorder, Immunological Disease” (*p*-score = 37 with 21 focus molecules) (Figure 8). Also, the *PRR32* gene was one of the focus molecules under “Cell Morphology, Embryonic Development, Hair and Skin Development and Function” (*p*-score = 19 with 13 focus molecules) (Figure 9).

CHRNE is a transmembrane acetylcholine receptor (AChR) and its mutations lead to congenital myasthenic syndrome which is responsible for perturbation on calcium signaling and post-synaptic Ca^2+^ accumulation (Zayas et al., 2007; Shen et al., 2018). Along with calcium voltage-gated channel alpha subunits (CACNA1D, CACNA1G, and CACNA2D2 in Figure 8), the *CHRNE* expression may induce normal calcium flux. Subsequently, activation of AMPK via phosphorylation by calcium sensitive kinase CAMKK2 (Herzig and Shaw, 2018) may lead to prevention of cardiomyocyte injury in the atrial appendage, as shown in AMPK-mediated prevention of cellular injury (Zhao et al., 2013; Bi et al., 2015).

PRR32 is one of the proline-rich proteins that have been recognized as tanning-binding salivary proteins which counteracts a negative effect of a phenolic compound, tannin, in mammalian herbivores (Shimada, 2006). Although *PRR32* expression was AA-specific in this current study, the role of *PRR32* is yet to be investigated in the heart, and also the relationship between *PRR32* and *ESR1* has not been established according to the network analysis (Figure 9). Estrogen receptor 1 (ESR1) is a nuclear receptor of 17beta-estradiol and ESR1 activation in animal models exhibited anti-inflammatory effects (Puzianowska-Kuznicka, 2012). ESR1 shared one toxicity-related list, “Cardiac Hypertrophy,” with proinflammatory cytokine tumor necrosis factor (TNF) (Figure 9). Because a relationship between ESR1 and TNF has also not been established (Figure 9) but expression of TNF in the heart leads to cardiac pathogenesis, the role of *ESR1* and *PRR32* will need to be further investigated. Additionally, network analyses were conducted for three unreported common-specific/enhanced genes, and results were presented in Supplementary Figures S4–S6. Mechanistic insights on these unreported genes were provided in the “Discussion” section.

## Discussion

The comprehensive human tissue transcriptome provided by the ongoing GTEx project was used to screen human heart-related genes including heart-specific genes, heart-enhanced genes, and DEGs, based on our previously developed method (Ahn et al., 2019). These transcriptomic data enabled large-scale identification of novel heart-specific/enhanced genes for further investigation on evidence of their relation to heart diseases and heart-related phenotypes. These approaches combined with pathway analysis provided a robust and integrative strategy for the consolidation of heart-related functions and diseases.

In this study, more than 70% of genes specific/enhanced to atrial appendage (32 out of 45) and left ventricle (32 out of 44) were overlapping genes, suggesting those common heart- specific/enhanced genes are involved in essential biological functions of the heart. According to our literature mining, the majority of those common-specific/enhanced genes have been reported in PubMed in relation to their functions and associated diseases in the heart; however, among the 32 common-specific/enhanced genes, three genes (*FBXO40*, *RD3L*, and *SMCO1*) have not been reported regarding the heart. Among non-overlapping AA-specific/enhanced protein-coding genes, four genes were unreported genes (*SBK2*, *PRR32*, *SBK3*, and *CHRNE*). “Diseases and Biological Functions” enriched with common-specific genes, AA-, and LV-specific/enhanced genes were apparently similar, but their canonical pathways showed differences regarding the significance in prediction of activation or inhibition and participating genes in the pathways. Since our gene sets were highly specific, but relatively small, many of the pathway genes might be excluded from the analysis. However, two distinct upstream genes of canonical pathways were found to be AA-specific/enhanced (*BMP10* and *FGF23*), and differences in downstream molecules were detected which may provide characteristics of the atrial appendage and left ventricle.

In order to increase specificity in either the atrial appendage or left ventricle, we matched specific/enhanced genes with DEGs and found unreported AA-specific and differentially upregulated three genes (*PRR32*, *SBK2*, and *CHRNE*). The epsilon subunit of acetylcholine receptor, CHRNE, is a transmembrane protein and mutations in *CHRNE* have been associated with congenital myasthenic syndrome which is characterized by compromised neuromuscular transmission and muscle weakness (Shen et al., 2018). This syndrome perturbs the kinetics of synaptic currents, leading to post synaptic Ca^2+^ accumulation (Zayas et al., 2007). The neurotransmitter, acetylcholine, prevents hypoxia-induced cellular injury by activating AMP-activated protein kinase (AMPK) (Zhao et al., 2013; Bi et al., 2015). AMPK is activated via phosphorylation by calcium sensitive kinase CAMKK2 in response to calcium flux, thus calcium signaling is linked to the regulation of energy metabolism by AMPK (Herzig and Shaw, 2018). Therefore, CHRNE/calcium/AMPK signaling cascades may play a role in preventing cardiomyocyte injury in AA, along with calcium voltage-gated channel alpha subunits (CACNA1D, CACNA1G, and CACNA2D2) shown in Figure 8. Also, activation of AMPK via phosphorylation by calcium sensitive kinase CAMKK2 has been reported to be independent from upstream kinases for AMPK (Herzig and Shaw, 2018).

Regarding a tannin suppressant PRR32, ESR1 activation in animal models of cardiac ischemia showed cardioprotective roles of ESR1 including reduction of inflammation, oxidative stress, and apoptosis of cardiomyocytes (Puzianowska-Kuznicka, 2012), but currently identified relations between *ESR1* and *PRR32* are inconsistent according to the network analysis. In addition, although ESR1 might have anti-inflammatory effects, a relationship between *ESR1* and *TNF* is also not established based on the network. TNF was linked to three toxicity-related lists generated by IPA: “Cardiac Fibrosis,” “Cardiac Hypertrophy” and “Cardiac Necrosis/Cell Death” and among them “Cardiac Hypertrophy” was shared between TNF and ESR1. Because expression of proinflammatory cytokine tumor necrosis factor (TNF) in the heart leads to cardiac pathogenesis and congestive heart failure, and therefore attenuation or inhibition of TNF expression has been investigated as clinical strategies (Feldman et al., 2000; Hartman et al., 2018), the unknown function of ESR1 and PRR32 gains attention. In addition, SBKs are kinases bind to the Src homology 3 (SH3) domain which is involved in various cellular functions such as cell proliferation, cytoskeletal modifications, and signal transduction (Kurochkina and Guha, 2013). In this study, *SBK2* was more AA-specific than *SBK3*, but pathways related to *SBK2* were not identified. The specific roles of these kinases in the atrial appendage are yet to be investigated.

Among the unreported common-specific/enhanced genes, F-box protein 40 (*FBXO40*) encodes a protein that forms SCF ubiquitin ligase complex, with SKP1 and Cullin proteins, which is involve in protein ubiquitination (Jin et al., 2004). An isoform of heat shock protein 90 (HSP90AA1), that was linked to FBXO40 (Supplementary Figure S4), plays roles in degrading proteins as a molecular chaperone and stabilizing TGF-beta signaling cascade (Garcia et al., 2016). The interaction between *FBXO40* and *HSP90AA1* may occurred through the ubiquitin ligase complex (Ehrlich et al., 2009), but their direct roles in the heart need further investigation. Another common-specific gene, retinal degeneration 3 like (*RD3L*), encodes a domain of retinol degeneration (RD) 3 protein which is associated with retinal degeneration (Friedman et al., 2006). The function of *RD3L* remains unknown in the heart, despite its specific cardiac expression shown in this study. In the network analysis, relations were found between *RD3L* and heat shock protein family A (Hsp70) member 9 (*HSPA9*) and between *RD3L* and WD repeat containing, antisense to TP73 (WRAP73), but the directions of influences between each other (activation or inhibition) were not predicted (Supplementary Figure S5). Among them, the *HSPA9* gene encodes a mitochondrial chaperone and its mutations were identified in patients with cardiac malformation (Royer-Bertrand et al., 2015), and this gene was linked to eNOS signaling which has a protective function in the cardiovascular system by regulating the diameter of blood vessels (Daiber et al., 2019) (Supplementary Figure S5). However, the relation was not statistically significant. In addition, single-pass membrane protein with coiled-coil domains 1 (*SMCO1*) was related to a transcription factor, cAMP-responsive element-binding protein (CREB), without prediction on the direction of influence (Supplementary Figure S6). The role of *SMCO1* is largely unknown, but its unknown interaction with CREB1 might be important because CREB1 is implicated in the regulation of cardiac pathophysiology (Matus et al., 2007).

Among identified DEGs between AA and LV, *BMP10*, *HAND1, MYL3*, and *XIRP2* were significantly enriched with “Morphogenesis of Cardiac Muscle” under the first-ranked “Cardiovasuclar System Development and Function, Embryonic Development, Organ Development” network. The expression of *BMP10* was upregulated in AA and the other three genes were upregulated in LV. The *BMP10* gene has been reported as the most highly expressed gene in the right atrial appendage compared to the left atrial appendage (Kahr et al., 2011) and the GTEx sampling site was the right atrial appendage. The AA-specific expression of the *BMP* gene in our results further revealed its specificity in the right atrial appendage compared to all other analyzed tissues including LV. It was also consistent with previous studies that reported HAND1 is a transcription factor whose expression is restricted to the left ventricle and contributes to chamber formation (Huang et al., 2004; Firulli et al., 2010). The *MYL3* gene is one of the cardiac sarcomeric genes and encodes the ventricular form of myosin light chain (Cohen-Haguenauer et al., 1989). Xin actin binding repeat containing 2 (XIRP2) is localized to the intercalate disks (ICDs) in cardiac muscle and is an actin-binding protein that interacts with actin filaments through conserved Xin repeats. XIRP2 may regulate cardiac ion channel by modulating the actin cytoskeleton, and its deficiency has been shown to be associated with cardiac conduction (Wang et al., 2014; Huang et al., 2018). Another significant subnetwork, “Hypertrophy of Left Ventricle” involved *NPPA* and *REN* (up-regulated in AA) and *IRX4* and *MYL3* (up-regulated in LV). The renin (*REN*) gene encodes protease renin which plays a central role in regulating blood pressure, and activation of the renin-angiotensin system is linked to the development of left ventricular hypertrophy (Cowan and Young, 2009). The role of the iroquois homeobox 4A (*IRX4)* gene has been reported in regulating chamber-specific expression of myosin isoforms and ventricular differentiation in animal models, and potential causal effects of its mutation in a ventricular septal defect in humans have been proposed (Bao et al., 1999; Bruneau et al., 2001; Cheng et al., 2011). As mentioned earlier, *NPPA* forms a cluster with *NPPB* and encodes a peptide hormone, but *NPPA* was more specific to AA compared to *NPPB*. The *MYL3* gene was both up-regulated in DEG in the LV and LV-specific gene, indicating its role as a left ventricle-specific myosin protein. Also, a canonical pathway that was predicted to be activated the most within the first-ranked network was “Calcium Signaling.” Considering the most enriched canonical pathways of the heart-specific/enhanced gene were not predicted to be activated or inhibited with DEGs, functional roles of the gene sets of heart-specific/enhanced genes and DEGs may be distinctive.

Taken together, this study reports on comprehensive identification of heart-specific genes, heart-enhanced genes and DEGs, and their relations with pathways associated with heart-related traits and diseases. Limitations to the present study that needs to be addressed include age of donors and stringent selection criteria. Given the donors aged 21–70 for the GTEx project, our identification of heart-specific genes and DEGs was limited to the adult stage. Thus, potentially important developmental gene expression in earlier stages could not be examined. In addition, we cannot rule out that some potentially important heart-related genes might not be incorporated in the current study due to stringent selection criteria (median 5-fold; FDR < 0.01); however, heart-specific genes identified in this study will need to be prioritized for further functional evaluations. In conclusion, our findings on novel heart-specific/enhanced genes and their functional implications, together with their pathways, further provide new insights into biological underpinnings of cardiac symptoms and diseases. To enhance our understanding on the genetic architecture underlying cardiac physiology and pathophysiology, continued attempts need to be made on further elucidation of functional aspects of those heart-specific genes while ultimately providing potential targets for cardiac gene therapy.

## Data Availability Statement

All datasets generated for this study are included in the article/Supplementary Material.

## Author Contributions

KL conceived the study. JA, HW, and KL developed the study designed and analyzed and interpreted the data. JA and HW performed the data processing and statistical analysis and drafted and wrote the manuscript. JA conducted the pathway analysis. All authors read, edited, and approved the final manuscript.

## Conflict of Interest

The authors declare that the research was conducted in the absence of any commercial or financial relationships that could be construed as a potential conflict of interest.

## Footnotes

1 https://www.qiagenbioinformatics.com/products/ingenuity-pathway-analysis

2 https://www.gtexportal.org/home

3 https://www.gtexportal.org/home/documentationPage

4 https://CRAN.R-project.org/package=dplyr

5 http://amp.pharm.mssm.edu/Enrichr

6 https://github.com/wdecoster/enrichr_cli

7 https://www.ensembl.org/info/data/ftp/index.html

8 https://CRAN.R-project.org/package=heatmap3

9 https://www.ncbi.nlm.nih.gov/pubmed/

10 https://CRAN.R-project.org/package=easyPubMed

11 https://CRAN.R-project.org/package=pubmed.mineR

12 https://www.gtexportal.org/home/documentationPage

13 https://www.gtexportal.org/home
