# Supplementary material for: Integrative Analysis Revealing Human Heart-Specific Genes and Consolidating Heart-Related Phenotypes
Source: Front Genet. 2020 Jul 30;11:777. doi: 10.3389/fgene.2020.00777 (PMC7438927; doi:10.3389/fgene.2020.00777)
Supplement: Supplementary file 1 [file Data_Sheet_1.pdf]

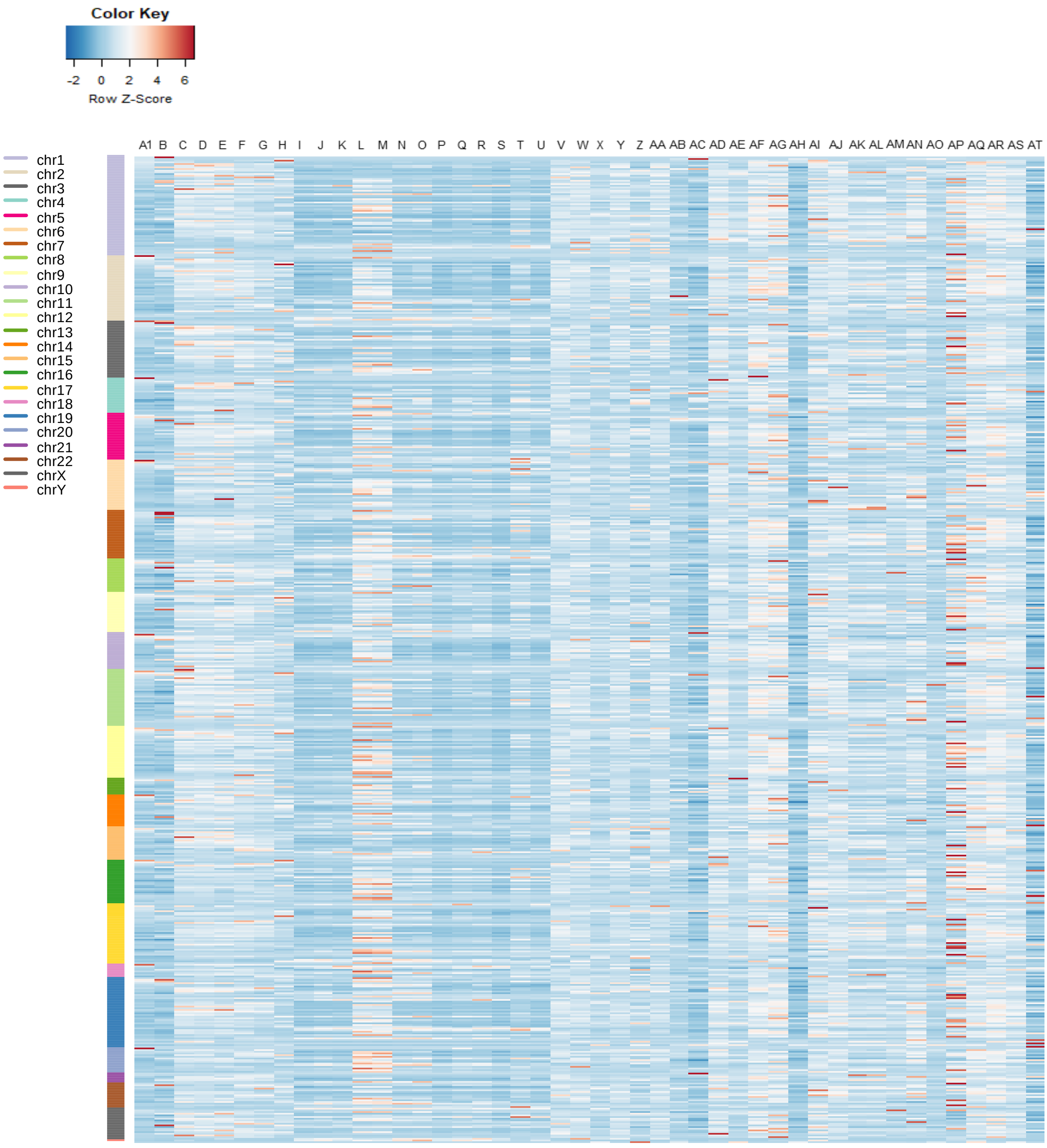

Supplementary Figure S1. Heat map of the 16,480 genes (in Fig. 1A) divided by chromosomes. Genes in each chromosome are ordered by RMVs (high to low) in atrial appendage (A1). Z-scores for RMVs of each row (i.e., gene) were calculated by heatmap3. |Z-score| ≥ 2 indicates greater than or equal to 2 standard deviation away from the mean of each row. Testis (AP) appears to show the greatest tissue-specificity as reported previously (Uhlén et al., 2015, Science. 347(6220):1260419).

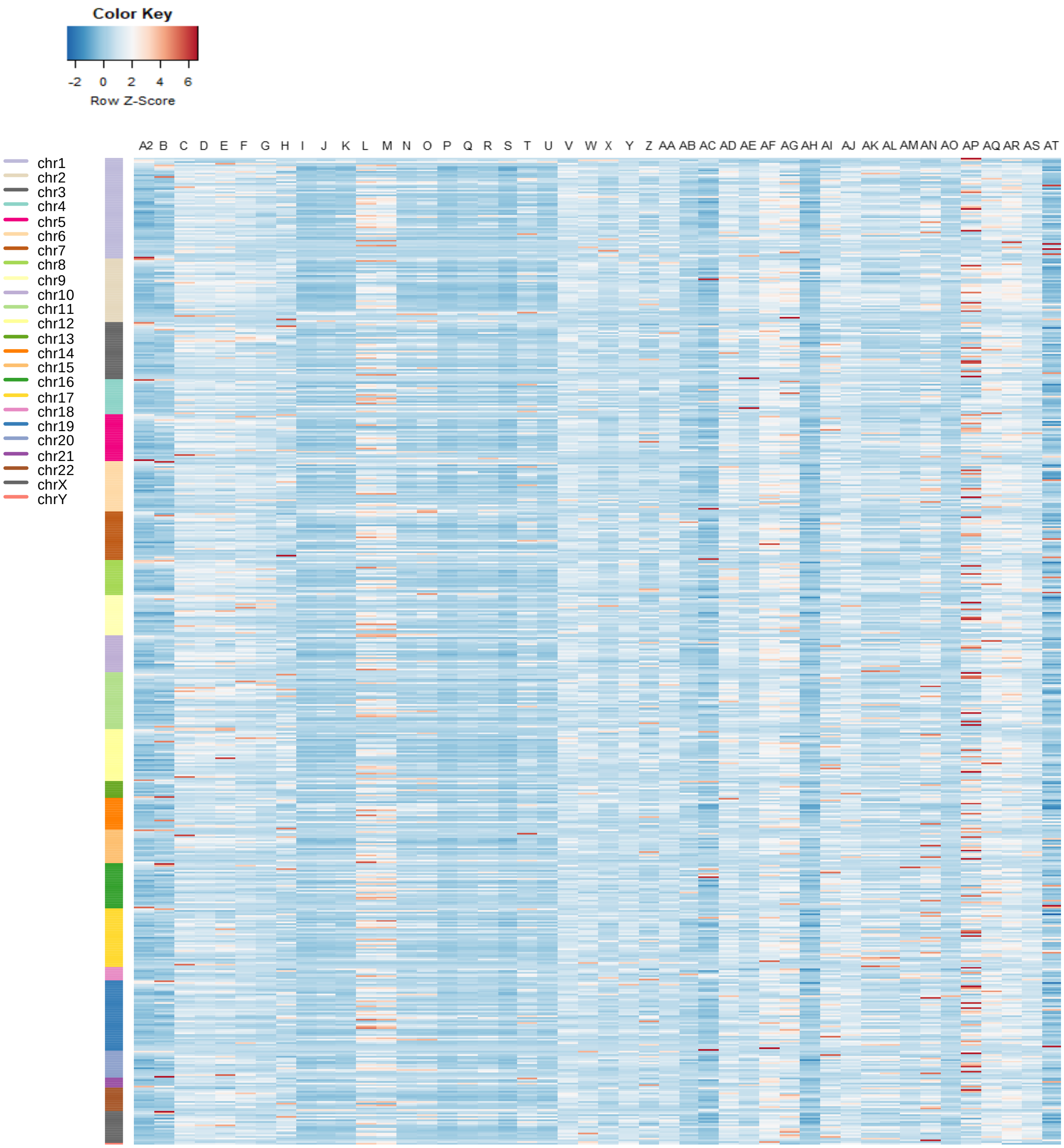

Supplementary Figure S2. Heat map of the 14,911 genes (in Fig. 1A) grouped by chromosomes. In each chromosome, genes are ordered by RMVs (high to low) in left ventricle (A2). Interpretation of row z-score and tissue-specificity of testis (AP) are addressed in Supplementary Figure S1.

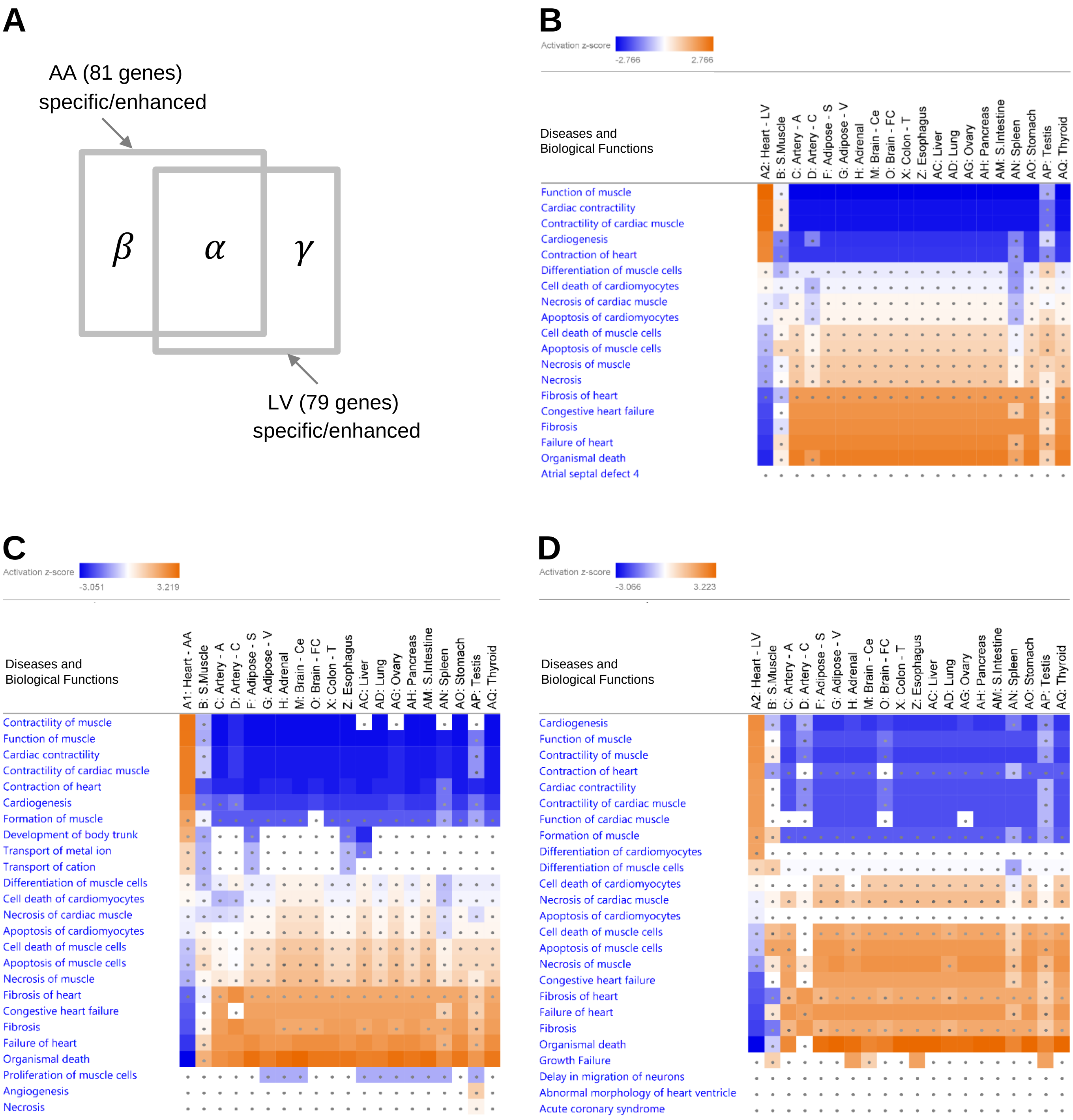

Supplementary Figure S3. IPA Comparison Analysis of heart-specific/enhanced genes. (A) Input genes for each analysis are shown as an intersection of 53 common-specific/enhanced genes (α), 81 AA-specific/enhanced genes (α + β), and 79 LV-specific/enhanced genes (α + γ). (B-D) Heat maps displayed 'Diseases and Biological Functions' that were enriched with common-specific/enhanced genes that were compared between LV and other tissues (B), AA-specific/enhanced genes (C), and LV-specific/enhanced genes (D). Details about inputs and activation z-scores in Figure 4.

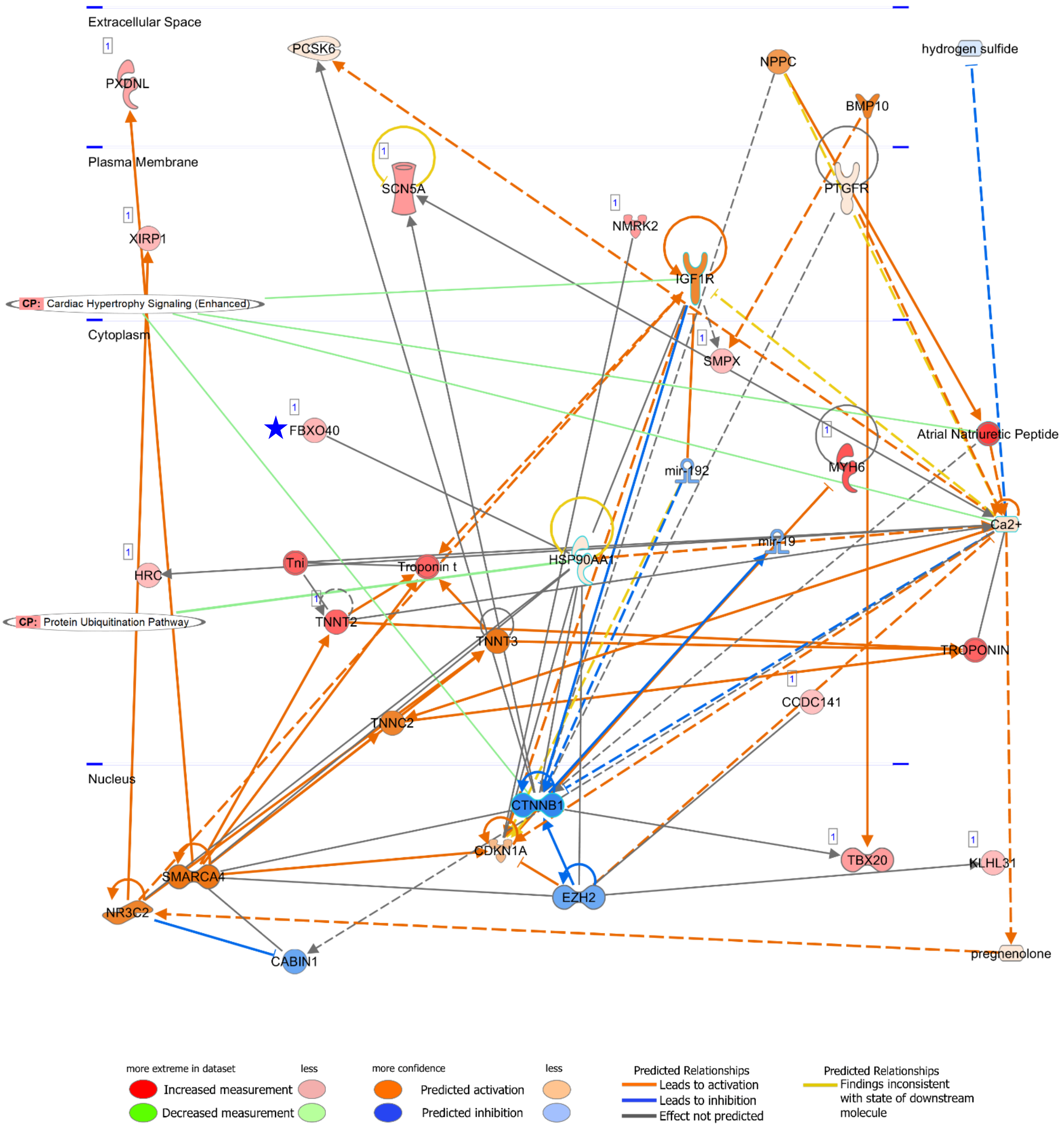

Supplementary Figure S4. A network analysis of a common heart-specific/enhanced gene, *FBXO40*, which is unreported regarding the heart. *CP*, canonical pathway. Details regarding the network in Figure 7C.

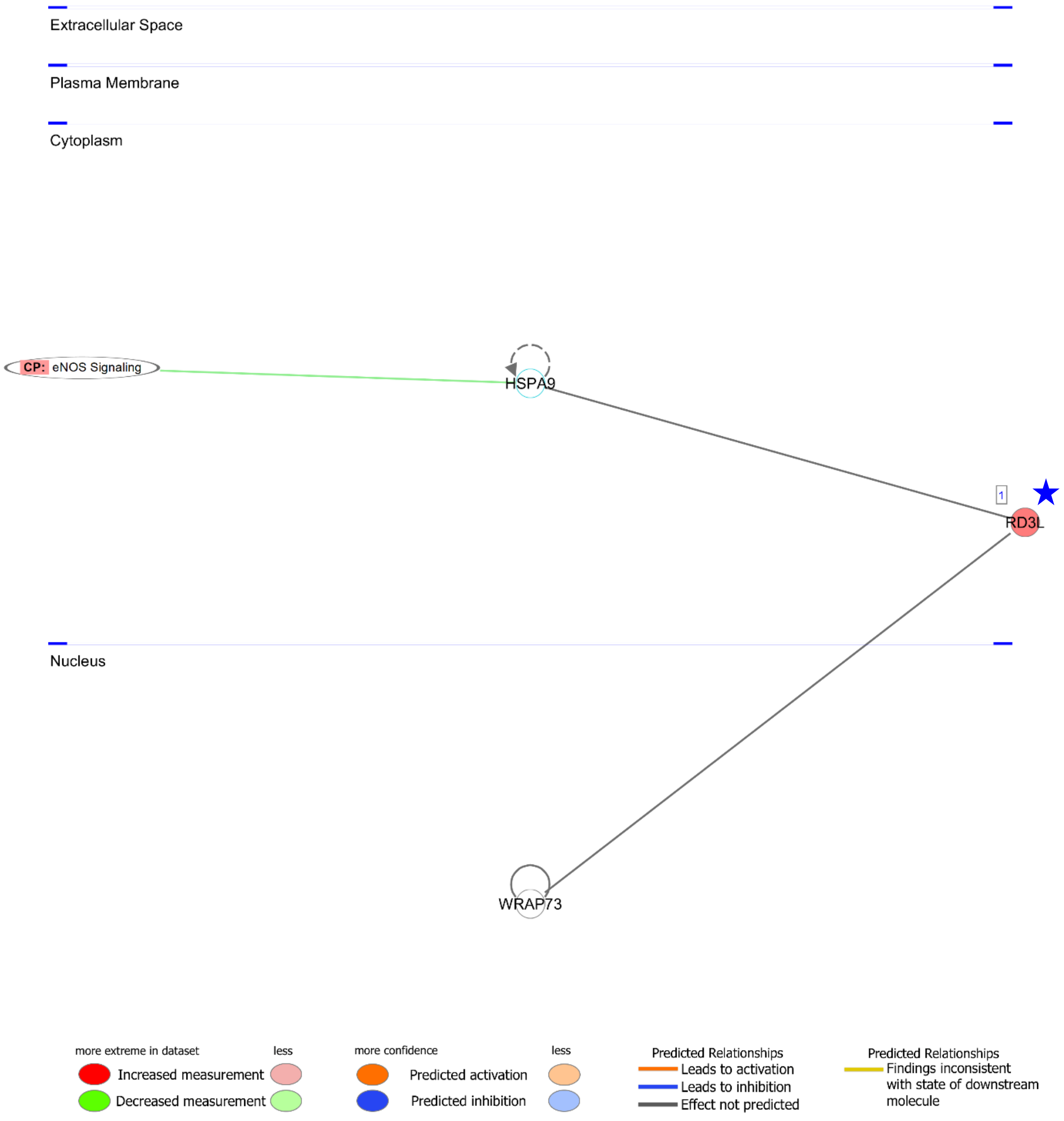

Supplementary Figure S5. Unreported common heart-specific/enhance gene, *RD3L*, and its related network. A canonical eNOS signaling is linked to a heat shock protein-encoding gene (*HSPA9*). *CP*, canonical pathway. Details regarding the network in Figure 7C.

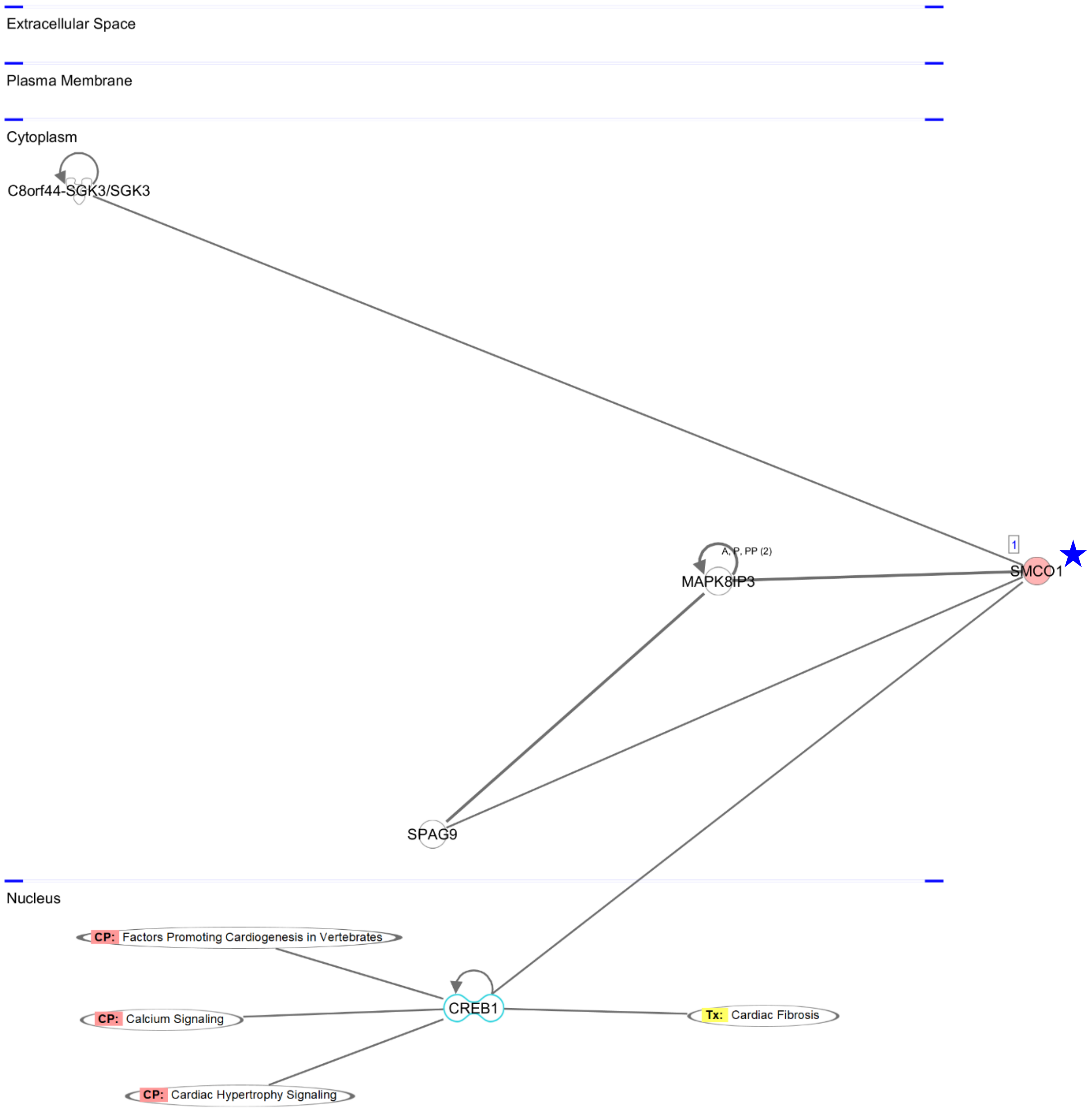

Supplementary Figure S6. Network of *SMCO1* which is a common heart-specific gene that has not been reported regarding the heart. *CP*, canonical pathway. *Tx*, toxicity-related lists. Details regarding the network in Figure 7C.
